# Glucose and Fructose Have Sugar-Specific Effects in Both Liver and Skeletal Muscle *In Vivo*: A Role for Liver Fructokinase

**DOI:** 10.1371/journal.pone.0109726

**Published:** 2014-10-17

**Authors:** Josep M. Fernández-Novell, Laura Ramió-Lluch, Anna Orozco, Anna M. Gómez-Foix, Joan J. Guinovart, Joan E. Rodríguez-Gil

**Affiliations:** 1 Dept. Biochemistry and Molecular Biology, University of Barcelona, Barcelona, Spain; 2 Dept. Animal Medicine and Surgery, Autonomous University of Barcelona, Bellaterra, Spain; 3 Centro de Investigación Biomédica en Red de Diabetes y Enfermedades Metabólicas Asociadas (CIBERDEM), Barcelona, Spain; 4 Institute for Research in Biomedicine, Barcelona Science Park, Barcelona, Spain; University of Navarra School of Medicine and Center for Applied Medical Research (CIMA), Spain

## Abstract

We examined glucose and fructose effects on serine phosphorylation levels of a range of proteins in rat liver and muscle cells. For this, healthy adult rats were subjected to either oral glucose or fructose loads. A mini-array system was utilized to determine serine phosphorylation levels of liver and skeletal muscle proteins. A glucose oral load of 125 mg/100 g body weight (G 1/2) did not induce changes in phosphorylated serines of the proteins studied. Loading with 250 mg/100 g body weight of fructose (Fr), which induced similar glycemia levels as G 1/2, significantly increased serine phosphorylation of liver cyclin D3, PI3 kinase/p85, ERK-2, PTP2 and clusterin. The G 1/2 increased serine levels of the skeletal muscle proteins cyclin H, Cdk2, IRAK, total PKC, PTP1B, c-Raf 1, Ras and the β-subunit of the insulin receptor. The Fr induced a significant increase only in muscle serine phosphorylation of PI3 kinase/p85. The incubation of isolated rat hepatocytes with 10 mM glucose for 5 min significantly increased serine phosphorylation of 31 proteins. In contrast, incubation with 10 mM fructose produced less intense effects. Incubation with 10 mM glucose plus 75 µM fructose counteracted the effects of the incubation with glucose alone, except those on Raf-1 and Ras. Less marked effects were detected in cultured muscle cells incubated with 10 mM glucose or 10 mM glucose plus 75 µM fructose. Our results suggest that glucose and fructose act as specific functional modulators through a general mechanism that involves liver-generated signals, like micromolar fructosemia, which would inform peripheral tissues of the presence of either glucose- or fructose-derived metabolites.

## Introduction

The rapid, relatively recent dietary changes in Western societies have highlighted the effects of ingesting certain sugars. Thus, substitution of glucose by other glucide sweeteners, such as fructose, has revealed unexpected side-effects, like transient uric acid peaks in serum [1], elevated cholesterol, lipid dysregulation [2], leptin resistance [3], inflammatory responses in tissues like the kidney [4], and hypertriglyceridemia and metabolic syndrome associated with insulin resistance [5]–[7]. The differences between glucose and fructose are attributed to the distinct ways in which they are metabolized in the liver. Thus, while glucose is metabolized in hepatocytes via glucose 6-phosphate (G 6P), mainly through the hexokinase-I/glucokinase pathway, fructose is metabolized via fructose 1-phosphate (F 1P), mainly through the fructokinase (FK) pathway [8], [9]. These distinct pathways lead to specific changes in intracellular levels of crucial regulatory metabolites, such as G 6P, glycogen and ATP, thus inducing sugar-specific changes in the rhythm of metabolic pathways like gluconeogenesis, glycogenesis, lipid synthesis and urea metabolism [8], [9]. These changes, in turn, lead to alterations in serum concentrations of metabolic products, such as triglycerides and uric acid [1], [2].

However, the difference in serum metabolite levels after liver metabolization of glucose and fructose are not the only way through which these sugars exert their specific *in vivo* effects. In fact, alone, these sugars act directly on key points of the regulatory function of several cells and tissues. Moreover, some of these actions do not appear to be related to the modulation of the expression of certain genes but are linked to specific changes in the general actions of proteins present in the cell, thus inducing rapid effects that trigger further physiological alterations in target tissues. As an example, glucose potentiates vascular smooth muscle cell chemotaxis through a phosphatidylinositol 3-kinase and mitogen-activated protein kinase-dependent up-regulation of the platelet-derived growth factor receptor [10]. Another example is the glucose-mediated activation of the protein kinase B (Akt) and the subsequent up-regulation of TGF-β1 in mesangial cells through a pathway that is dependent on the activity of the protein kinase C-β1 [11]. Likewise, one of the key signals that induces pancreatic insulin secretion is linked to the glucose-induced tyrosine phosphorylation of protein p125 in beta cells and pancreatic islets [12]. Fructose also has specific effects on the activity of certain proteins. An example is the fructose-induced insulin resistance in adipose tissue, which is linked to activation of the protein kinase C [13]. Other well known fructose-induced alterations in insulin-sensitive tissues are associated with changes in cell signalling and inflammatory pathways through modification of the activity of proteins like tumor necrosis alpha (TNFα), c-Jun amino terminal kinase 1 (JNK 1), protein tyrosine phosphatase 1B (PTP-1B), liver X receptor, farnesoid X receptor and sterol regulatory element-binding protein-1c (SREBP-1c; see 6). However, little is known about the mechanism/s underlying the origin of these effects and their *in vivo* regulation by the liver.

The liver is the main regulatory tissue of sugar metabolism and the subsequent utilization of these molecules in the body. Here we aimed to establish a unified view of the differential effects of the *in vivo* administration of glucose and fructose on this organ. For this purpose, healthy adult rats were subjected to an oral load of either fructose or glucose at a range of doses. Using a mini-array system, we then analyzed the phosphorylation levels of proteins linked to cell cycle control, phospho-dephosphorylation regulatory mechanisms, and cell-to-cell interactions in liver and in the most important peripheral tissue with respect to sugar consumption, such as skeletal muscle. Furthermore, a similar analysis was performed in isolated hepatocytes from starved healthy rats and from cultured rat skeletal muscle L6E9 cells incubated with fructose or glucose, in order to gain further insight into the nature of the changes induced by these sugars in these cell types.

## Materials and Methods

### Animals and *in vivo* treatment protocols

All animal procedures and treatment protocols were in accordance with the Law 5/1995 on Animal Protection for Research and other Scientific Purposes (Generalitat de Catalunya, Spain). From this Law, the University of Barcelona developed its own ethical protocols guidelines that follow all of the international ones. Under this basis, the animal procedures and treatment protocols utilized in this work have been specifically approved by the Institutional Animal Care and Use Committee (IACUC) of the University of Barcelona. Following this specific approval, healthy adult male Wistar HsdOla:WI rats of 8 weeks of age as a mean weight of 200 g and without any genetic modification or other previous manipulations were utilized. Animals were housed in a SPF facility [Plataforma de Recerca Aplicada a l’Animal de Laboratori (PRAAL-PCB); Barcelona Science Park, University of Barcelona] in which they were housed in polysulfone Type III shoebox cages (Tecniplast; Varese, Italy). Bedding material used was autoclaved aspen wood. Rats were house 2 each per cage. Animals were kept under a constant 12 h–12 h light-dark cycle at 20°C-22°C and at a relative humidity of 50%–60%. They were allowed to eat and drink *ad libitum*. All animals were health- and welfare-assessed daily. Prior to, during and after the experiments further welfare assessment was performed by researchers and technicians of the facility. Animals were fasted 24 h before the start of the *in vivo* oral sugar load. The final step of the experimental study was in all cases carried out between 9∶00 AM and 11∶30 AM. This final step was performed at the facilities of the PRAAL-PCB. Animals were then divided into 6 groups. The first group (Control) was immediately anesthetized with halotan in a specifically designed chamber to obtain a halotan saturated atmosphere and subsequently killed by decapitation. Halotan was selected because being a potent anaesthesic that completely avoided any subsequent pain of suffering of animals inducing minimal effects on the evaluated parameters. The other 5 groups were intragastrically intubated and administered a 5 mL solution as follows:

Saline group: 0.9% (w/v) NaCl solution (saline solution).
Glucose 1/4 group (G 1/4): 65 mg/100 g body weight of glucose in saline solution.
Glucose 1/2 group (G ½): 125 mg/100 g body weight of glucose in saline solution.
Glucose group (G): 250 mg/100 g body weight of glucose in saline solution.
Fructose group (F): 250 mg/100 g body weight of fructose in saline solution.

In each case, animals from the same experimental group were caged together with a total of 2 animals per cage. Intragastrical intubation was immediately removed after administration of the solutions and animals were returned to their cages for 30 min. They were then anesthetized with halotan and killed by decapitation as previously indicated. Immediately before the intragastric load, an aliquot of blood of approximately 10 µL was taken from each animal through tail puncture in order to measure glycemia. Immediately after decapitation, blood was collected in heparinized tubes while liver and longissimus dorsi muscle samples were dissected and frozen in liquid N_2_ for further processing. Each experimental group was composed by 5 rats. This number was established following our previous experience with other experimental designs based in the obtainment of information through a mini-array system in which the same number of separate replicates was utilized as a minimally significant to yield relevant results [14]. Thus, the total number of animals utilized for the *in vivo* experimental procedure was of 30. In all cases, animals were processed during the final steps of the experimental procedure following a random order of processing.

### Preparation and incubation of hepatocytes

Hepatocyte suspensions were prepared from healthy adult male Wistar HsdOla:WI rats of 8 weeks of age and weighted 200 g that were previously fasted for 24 h, as described in [15]. The total number of animals utilized for this purpose was of 5. In this way, the total number of animals utilized in this work after summing those utilized in the *in vivo* experimental procedure and those used for the isolation of hepatocytes was of 35. Cells were resuspended in Krebs-Ringer bicarbonate buffer (pH 7.4). Samples (4 mL) of these suspensions, containing 4–5×10^6^ cells/mL, were incubated at 37°C with gassing and continuous shaking (100 strokes/min). Glucose and fructose were dissolved in a saline solution at a concentration of 1 M. Suitable volumes of these solutions were added to the cell suspensions to obtain the desired final concentration (10 mM in all cases). Control hepatocytes were incubated with the same volume of saline solution. After 5 min of incubation, cells were centrifuged (3000 *g* for 20 s) and pellets were immediately frozen in liquid N_2_ until their subsequent processing.

### Preparation and incubation of cultured skeletal muscle cells

We utilized rat L6E9 myoblasts, which were kindly donated by Dr. Anna Gumà (University of Barcelona). Myoblasts were grown in DMEM medium (Invitrogen; Carlsbad, CA, USA) containing 25 mM glucose and supplemented with 25 mM 4-(2-hydroxyethyl)-1-piperazineethanesulfonic acid (HEPES) and 10% (w:v) fetal bovine serum (FBS). Cells were cultured until reaching confluence. They were then induced to fuse by means of incubation in DMEM medium containing 25 mM glucose, 25 mM HEPES and 2% (w:v) FBS for 5 days. Afterwards, the differentiated myotubes were then incubated for 18 h in DMEM medium with any addition before being used in the experiments. As in case of hepatocytes, the incubation of the differentiated myotubes with the utilized monosaccharides was of 5 minuts. Afterwards, cells were centrifuged (3000 *g* for 20 s) and pellets were immediately frozen in liquid N_2_ until their subsequent processing. The time of incubation of 5 minuts was chosen both in hepatocytes and myotubes as a good mean time in which any putative effect of sugars on phosphorylation levels can be easily detected, following all of the literature accumulated at this respect in the last 50 years.

### Mini-array analysis of selected proteins from tissue and cell extracts

**Figure 1:**
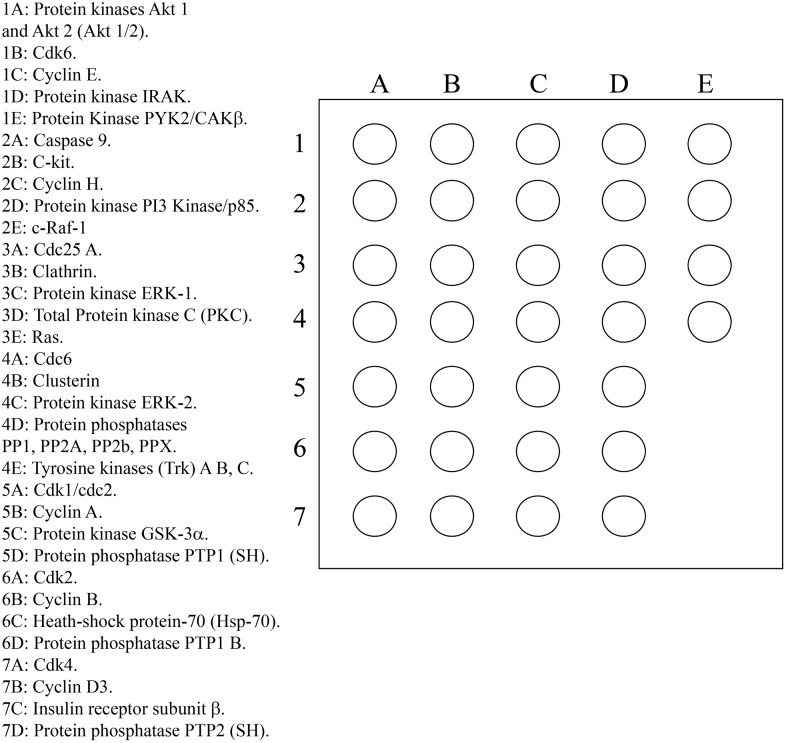
General scheme of protein distribution for the mini-array analysis.

Liver and muscle tissues, as well as resuspended hepatocytes samples were homogenized in 1 mL of an ice-cold extraction solution comprising a 15 mM Tris/HCl buffer (pH 7.5) plus 120 mM NaCl, 25 mM KCl, 2 mM EGTA, 2 mM EDTA, 0.1 mM DTT, 0.5% Triton X-100, 10 µg/mL leupeptin, 0.5 mM PMSF and 1 mM Na_2_VO_4_ (extraction solution) using a Polytron®. Homogenized samples were then left for 30 min at 4°C and then centrifuged at 10,000 *g* for 15 min at 4°C. Supernatants were taken and then used to test the degree of tyrosine, serine and threonine phosphorylation of selected proteins included in a Custom AntibodyArrayTM (Hypromatrix, Inc. Worcester, MA, USA), following the standard protocol provided by the manufacturer. Briefly, each sample was diluted in 2 mL of extraction solution containing 1% dry milk to reach a final protein concentration of 2 µg/µL. Simultaneously, Antibody ArrayTM membranes were placed in suspension culture dishes (60 mm×15 mm; Corning Incorporated; Corning, NY, USA) and incubated with a blocking solution containing 150 mM NaCl, 25 mM Tris and 0.05% (v:v) Tween-20 (TBST; pH 7.5) plus 5% (w:v) dry milk and left for 1 h at room temperature under slow shaking. Membranes were then incubated with the samples for 2 h at room temperature under slow shaking. After incubation, membranes were washed 3×15 min with TBST. They were then incubated with 10 µg/mL HRP-conjugated anti-phosphoserine antibody (HM2070; Hypromatrix). At the same time, preliminary experiments involving the determination of phosphotyrosine and phosphoserine phosphorylation levels of the proteins were performed. For this purpose, HRP-conjugated anti-phosphotyrosine (HM2040; Hypromatrix) and anti-phosphothreonine (HM2090; Hypromatrix) were also utilized. However, since the results obtained with the phosphotyrosine and phosphothreonine antibodies were unconsistent, they were not included in this work (data not shown). In all cases, antibodies were diluted in TBST and array membranes were subsequently incubated with these antibodies for 2 h at room temperature under slow shaking. Finally, samples were washed 3×15 min with TBST and then incubated with peroxidase substrate (ECL-Plus Western Blotting Detection; GE Healthcare UK Ltd, Buckinghamshire, England) and exposed to X-ray film (Amersham Hyperfilm ECL; GE Healthcare UK Ltd, Buckinghamshire, England). The total protein content of the supernatants was determined by the Bradford method [16] using a commercial kit (Bio-Rad Protein Assay Dye Reagent; Bio-Rad Laboratories). The intensity of the spots obtained was quantified using specific software for image analysis of blots and arrays (Multi Gauge v3.0; Fujifilm Europe; Düsseldorf, Germany), in which the background was previously standardized for all the arrays analyzed. The values obtained for the control samples, i.e. those incubated in the absence of sugars, were transformed in order to obtain a basal arbitrary value of 100, from which the intensity values for the other samples were calculated. Furthermore, two types of negative control were applied. In one, two arrays were incubated with a randomly chosen sample but without further incubation with the primary antibody. In the other, two arrays were incubated with the antibodies but without samples. The proteins included in this study are described in Fig. 1. All the antibodies included in the arrays were previously tested for their specificity for rat tissues by the manufacturer of the array.

### Analytical procedures in serum samples

Glycemia was measured by the hexokinase method and fructosemia by the fructokinase technique. Both determinations were performed using the appropriate commercial kits (Roche Biomedical; Basel, Switzerland). Serum insulin levels were determined by an enzyme-linked immunosorbent assay (ELISA) using a commercial kit (Crystal Chem, Chicago, ILL).

### Determination of ATP and G 6P in resupended hepatocytes

Frozen cellular pellets were homogenized in 1 mL 10% (v/v) ice-cold HClO_4_. Homogenates were centrifuged at 10,000 *g* for 15 min at 4°C and the resultant supernatants were neutralized with 5 M K_2_CO_3_ before analysis. G 6P and ATP were determined enzymatically as in [17] and [18], respectively.

### Statistical analysis

Statistical analyses were performed using SPSS 15.0 for Windows (SPSS Inc., Chicago, IL, USA) and data are presented as means±S.E.M. Data were tested for normality and homoscedasticity using the Shapiro–Wilk and Levene tests. When necessary, data were transformed using arcsine square root before a generalised estimating equation (GEE), an extension of generalised linear model (GLM) for repeated-measures, was performed. Characteristics of the GEE were normal distribution and identity link function. The inter-subject factor was treatment. In all cases, each sugar administration/incubation was the dependent variable and multiple post hoc comparisons were calculated using Sidak’s test. In all statistical analyses, the minimal level of significance was set at P<0.05. However, since the analytical technique has an intrinsic subjective component, we considered true differences to be only those for which P<0.05 and with a percent difference above 20%.

### Suppliers

All chemical reagents were of analytical grade and were obtained from Sigma (Saint Louis, MO), Merck (Darmstadt, Germany), Bio-Rad Laboratories (Hercules, CA) and EMS (Fort Washington, PA).

## Results

### Changes in serum levels of glucose, fructose and insulin and in serine phosphorylation levels of proteins linked to cell cycle regulation and phospho/dephosphor-ylation mechanisms in rats subjected to an oral glucose or fructose load

Oral administration of 250 mg of glucose/100 g body weight (G) induced an increase in glycemia from 5.9 mM±0.3 mM to 13.0 mM±1.1 mM after 30 min (Table 1). The administration of the same dose of fructose produced a lower increase (P<0.05) in glycemia, which reached values of 9.8 mM±0.9 mM after 30 min. Interestingly, the oral administration of 125 mg of glucose/100 g body weight (G 1/2) increased glycemia after 30 min to values similar to that reached with 280 mg of fructose/100 g body weight (9.5 mM±0.9 mM after glucose administration, see Table 1). Oral saline administration did not modify glycemia, thereby indicating that the values observed were not influenced by the administration technique used.

**Table 1:** Values of glycemia, fructosemia and insulinemia in rats subjected to either an oral glucose or fructose load.

	Control	Saline	Glucose 1/4	Glucose 1/2	Glucose	Fructose
Glycemia (mM)	5.9±0.3^a^	5.4±0.5^a^	7.0±0.4^b^	9.5±0.6^c^	13.0±1.1^d^	9.8±0.9^c^
Fructosemia (µM)	3.9±3.0^a^	3.9±3.5^a^	1.7±1.6^a^	1.1±1.1^a^	7.4±6.8^a^	73.8±7.0^b^
Insulinemia (mIU/L)	62.3±9.9^a^	84.1±9.6^a^	220.7±11.1^b^	217.4±10.8^b^	251.6±12.3^c^	193.0±11.2^b^

The experimental groups are described in the Materials and Methods section. Different superscripts in a row indicate significant (P<0.05) differences following the statistical analysis described in the Material and Methods section. Thus, data marked with an “a” in superscript are significantly different from those marked with any other letter in the same file. Results are expressed as means±S.E.M. for 5 individuals in each experimental group.

The oral administration of glucose did not induce measurable values of fructosemia at any of the loads tested. In contrast, the oral fructose loads caused fructosemia, which reached 73.8 µM±6.0 µM after 30 min (Table 1). Concomitantly, insulinemia reached maximal values 30 min after the glucose load (from 62.3 mIU±L9.9 mIU/L before administration to 251.6 mIU/L±12.3 mIU/L after loading of 250 mg/100 g body weight glucose, see Table 1). Fructose administration induced a lower increase in insulinemia than that caused by the maximal glucose load. However, insulinemia after fructose loading was not significantly different to that detected in animals after the administration of G 1/2 (193.0 mIU/L±11.2 mIU/L in rats receiving fructose vs. 217.4 mIU/L±10.8 mIU/L in those receiving G 1/2, Table 1).

**Figure 2:**
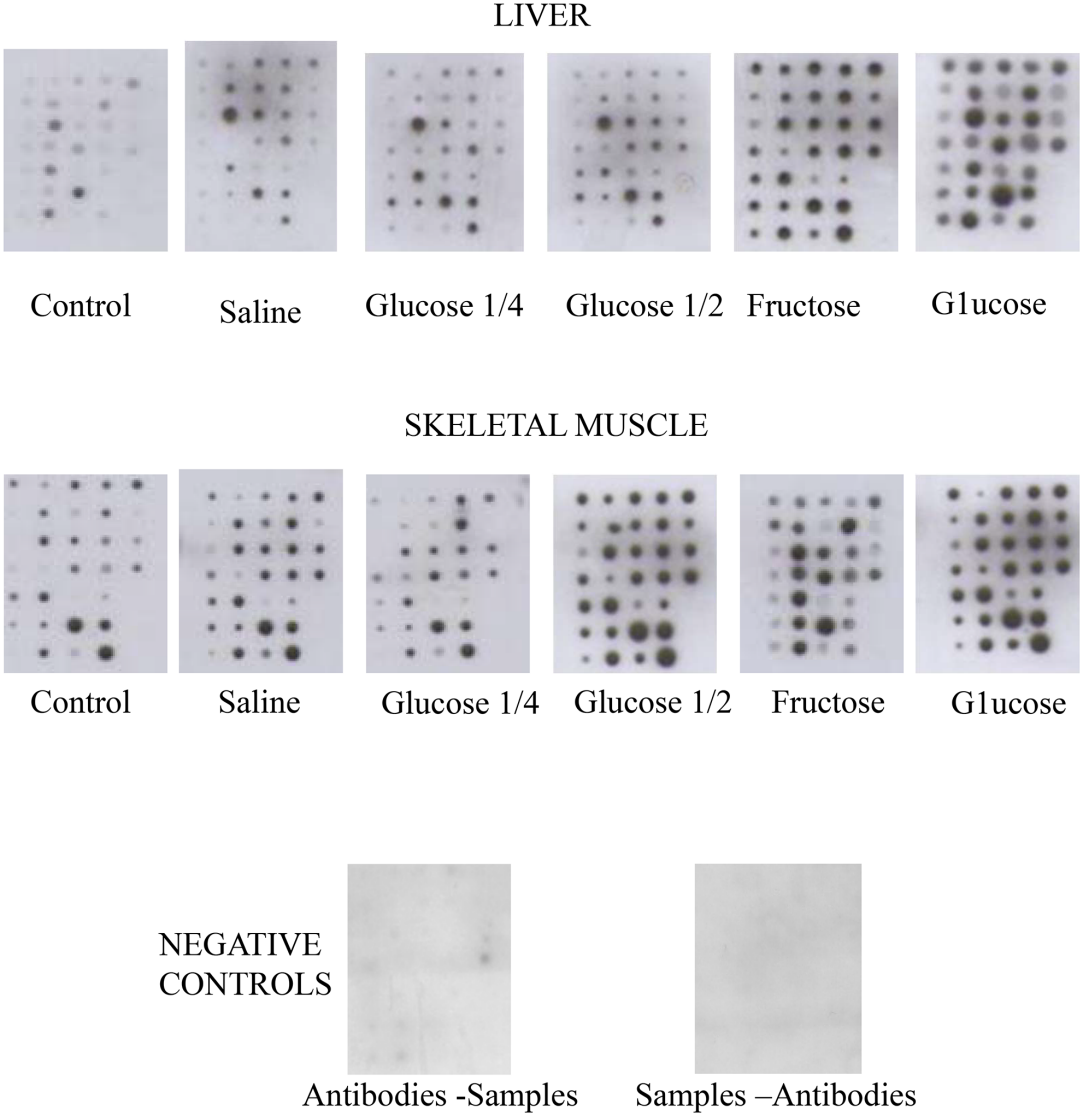
Mini-array analysis of the serine phosphorylation status of several proteins involved in overall cell function and the regulation of cell cycle in whole rat liver and skeletal muscle after oral loading of different amounts of glucose and fructose. The mini-array analysis and the distribution of the proteins are described in the Material and Methods section and Figure 1. Healthy adult rats were subjected to an oral glucose or fructose load, as described in the Material and Methods section. The serine-phosphorylation levels of each spot in the mini-arrays were then analyzed. Control: rats that were not subjected to an oral load. Saline: rats subjected to a mock oral load with xx mL of saline solution (0.9%; w/v, NaCl in fresh drinking water). Glucose 1/4: animals subjected to an oral load of 65 mg glucose/100 g body weight. Glucose 1/2: animals subjected to an oral load of 125 mg glucose/100 g body weight. Glucose: animals subjected to an oral load of 250 mg glucose/100 g body weight. Fructose: animals subjected to an oral load of 280 mg fructose/100 g body weight. C-: negative controls. Liver: whole liver extracts. Muscle: whole skeletal muscle extracts. The Figures shows a representative image for five separate experiments. Negative controls were designed as described in the Material and Methods section.

The oral administration of saline did not induce any significant change in the serine phosphorylation levels of the proteins compared to rats that received non-intragastric infusion. The minimal load of 65 mg/100 g body weight did not cause any significant change in the serine phosphorylation levels of any of the proteins studied (Fig. 2 and Table 2). In contrast, the maximal glucose load of 250 mg/100 g body weight caused a significant (P<0.05) increase in this parameter for liver total protein kinase C, ERK-2, protein phosphatase PTP1B, c-kit and clathrin, when compared with saline-treated rats (Fig. 2 and Table 2). Remarkably, the highest glucose load also induced a very intense and significant (P<0.05) increase in the serine phosphorylation levels of cyclin D3 (from 100.0±5.1 arbitrary units in saline-loaded animals to 180.9±6.4 arbitrary units in high glucose-loaded animals, see Fig. 2 and Table 2). The fructose load also induced an increase (P<0.05) in serine phosphorylation of several liver proteins, like ERK-2, PTP2, cyclin D3, PI3 kinase/p85 and clusterin (Fig. 2 and Table 2). However, of note, the highest glucose load increased serine phosphorylation of total PKC, PTP1B, c-kit and clathrin, while the highest fructose load increased that of PI3 kinase/p85, clusterin and especially PTP2 (Fig. 2 and Table 2). Remarkably, comparison of the oral treatments that rendered similar glycemia and insulinemia levels, namely the oral load of 125 mg of glucose/100 g body weight (G 1/2) and the oral fructose load, revealed a totally different behavior of the two experimental groups. Thus, the G ½ load did not induce any significant increase in the serine phosphorylation of any of the proteins analyzed. This finding clearly contrasted with that found for the fructose load, which, as indicated above, caused several significant (P<0.05) increases (i.e., PTP2 went from 100.0±4.0 arbitrary units after saline load to 107.7±5.3 arbitrary units after G1/2 glucose load and to 142.4±6.2 arbitrary units after fructose load, see Fig. 2 and Table 2).

**Table 2:** Relative increase in serine phosphorylation levels of selected liver proteins in rats subjected to either an oral glucose or fructose load.

	Control	Saline	Glucose 1/4	Glucose 1/2	Glucose	Fructose
Cyclin D3	96.1±4.9^a^	100.0±5.1^a^	96.6±4.7^a^	104.3±5.6^a^*	180.9±6.4^b^	147.2±6.7^c^*
PI3 kinase/p85	88.3±6.4^a^	100.0±7.1^a^	101.8±7.3^a^	117.3±7.6^a^	117.3±7.7^a^	123.9±7.7^b^
Total PKC	89.2±5.7^a^	100.0±6.2^a^	108.2±6.7^a^	121.0±7.0^a^	137.4±6.5^b^	121.0±5.3^a^
ERK-2	92.2±4.7^a^	100.0±5.1^a^	100.6±5.0^a^	121.7±5.8^a^	151.1±6.2^b^	127.8±6.0^b^
PTP1B	79.9±5.8^a^	100.0±5.9^a^	102.7±6.0^a^	104.5±5.7^a^	123.0±5.7^b^	107.2±5.7^a^
PTP2 (SH)	91.2±3.8^a^	100.0±4.0^a^	104.0±4.6^a^	107.7±5.3^a^*	120.1±5.9^a^	142.4±6.2^b^*
c-kit	98.0±3.3^a^	100.0±3.7^a^	103.0±3.2^a^	109.5±4.8^a^	131.1±4.7^b^	114.9±4.8^a^
Clathrin	78.6±4.2^a^	100.0±5.0^a^	100.6±4.9^a^	103.2±4.9^a^	134.1±5.6^b^	100.7±4.0^a^
Clusterin	93.5±4.0^a^	100.0±4.4^a^	86.9±4.7^a^	100.6±4.7^a^*	92.1±3.9^a^	130.4±4.9^b^*

Table shows proteins from arrays in which significant (P<0.05) differences above 25% were obtained compared to control or saline. Results are expressed in arbitrary units, transforming the intensity obtained to 100 in control points. Different superscript letters in a row indicate significant (P<0.05) differences among values from the same row. Thus, data marked with an “a” in superscript are significantly different from those marked with any other letter in the same file. Additionally, superscript asterisks indicate significant (P<0.05) differences between Glucose 1/2 values and Fructose ones. In both cases, differences were established following the statistical analysis described in the Material and Methods section. Acronyms for the proteins are described in Figure 1 while the experimental groups are described in the Materials and Methods section. Results are means±S.E.M. for 5 individuals in each group.

The effects of oral monosaccharide administration on serine phosphorylation levels of skeletal muscle proteins differed to those observed in liver. Again, the oral administration of either saline or 65 mg glucose/100 g body weight did not significantly modify any protein. Furthermore, the maximal glucose load induced an increase (P<0.05) in serine phosphorylation of muscle total PKC, PI3 kinase/p85, IRAK, PTP1B, cyclin H, Cdk2, c-Raf 1, ras, clusterin and the β subunit of the insulin receptor (Fig. 2 and Table 3). The administration of G1/2 glucose produced less intense effects, with a significant (P<0.05) increase (P<0.05) in muscle serine phosphorylation of cyclin H, Cdk2, IRAK, PTP1B, total PKC, c-Raf, ras and the β subunit of the insulin receptor. In contrast, the fructose load, which induced an increase in glycemia and insulinemia similar to those observed after the administration of the G1/2 load, caused a significant (P<0.05) increase only in the serine phosphorylation of muscle PI3 kinase/p85 (from 100.0±6.3 arbitrary units after saline administration to 136.6±6.4 arbitrary units after fructose treatment; see Fig. 2 and Table 3).

**Table 3:** Relative increase in serine phosphorylation levels of selected skeletal muscle proteins in rats subjected to an oral glucose or fructose load.

	Control (n = 5)	Saline (n = 5)	Glucose 1/4 (n = 5)	Glucose 1/2 (n = 5)	Glucose (n = 5)	Fructose (n = 5)
Cyclin H	110.7±4.9^a^	100.0±6.4^a^	109.3±5.1^a^	142.9±5.6^b^*	144.4±5.4^b^	109.4±6.1^a^*
Cdk2	106.8±6.6^a^	100.0±6.0^a^	105.9±6.5^a^	138.1±6.1^b^*	141.3±6.4^b^	99.8±5.7^a^*
IRAK	104.4±5.2^a^	100.0±5.5^a^	112.6±5.8^a^	127.4±5.6^b^*	138.4±5.3^b^	98.1±5.7^a^*
PI3 Kinase/p85	99.7±3.9^a^	100.0±6.3^a^	99.9±4.6^a^	117.6±6.9^a^*	123.7±6.5^b^	136.6±6.4^b^*
Total PKC	103.6±7.0^a^	100.0±5.7^a^	102.0±5.7^a^	128.3±5.3^b^*	140.6±6.0^b^	102.0±5.0^a^*
PTP1B	99.9±6.3^a^	100.0±4.6^a^	103.0±5.2^a^	131.7±5.4^b^*	138.2±6.1^b^	101.0±5.2^a^*
c-Raf 1	101.9±4.6^a^	100.0±4.2^a^	102.4±4.8^a^	141.8±5.3^b^*	151.0±5.7^b^	102.7±4.4^a^*
Ras	104.9±4.7^a^	100.0±5.2^a^	104.8±5.7^a^	132.0±6.9^b^*	150.5±5.1^b^	98.1±4.9^a^*
Clusterin	100.2±5.3^a^	100.0±5.5^a^	98.8±5.7^a^	109.2±5.8^a^	136.5±6.1^b^	123.1±6.6^a^
β subunit insulin receptor	106.0±5.2^a^	100.0±5.7^a^	104.3±5.6^a^	133.3±5.9^b^*	146.8±5.8^b^	108.6±5.2^a^*

Table shows proteins from arrays in which significant (P<0.05) differences above 25% were obtained. Results are expressed in arbitrary units, transforming the intensity obtained to 100 in control points. Different superscript letters in a row indicate significant (P<0.05) differences among values from the same row. Thus, data marked with an “a” in superscript are significantly different from those marked with any other letter in the same file. Additionally, superscript asterisks indicate significant (P<0.05) differences between Glucose 1/2 values and Fructose ones. In both cases, differences were established following the statistical analysis described in the Material and Methods section. Acronyms for the proteins are described in Figure 1 while the experimental groups are described in the Materials and Methods section. Results are means±S.E.M. for 5 individuals in each group.

### Changes in intracellular G 6P and ATP levels and in serine phosphorylation levels of proteins linked to cell cycle regulation and phospho/dephosphorylation mechanisms in isolated hepatocytes incubated in the presence of glucose or fructose

Isolated hepatocytes were incubated in media without sugars or in the presence of three distinct combinations of monosaccharides. The rationale behind these combinations is that in the previously described *in vivo* experimental design, hepatocytes were subjected to different concentrations of sugars depending on their specific location in the liver. Thus, after a glucose load all hepatocytes were exposed to a similar glucose concentration, which was the one determined by blood glucose levels. On the contrary, after the fructose load, liver hepatocytes were classified into two conditions. Those in contact with portal blood were incubated with about 10 mM fructose, while those in contact with peripheral blood were maintained under high concentrations of glucose (about 10 mM) and very low concentrations of fructose (about 75 µM). Thus, our next experimental design was based on the incubation of isolated hepatocytes from starved healthy rats in the presence of 10 mM glucose, 10 mM fructose or 10 mM glucose plus 75 µM fructose in order to mimic the range of conditions observed in hepatocytes *in vivo*.

The incubation of hepatocytes with 10 mM glucose for 5 min induced a significant (P<0.05) increase in the intracellular levels of both G 6P (27.9 nmol/mg protein±4.7 nmol/mg protein vs. 16.2 nmol/mg protein±3.4 nmol/mg in control cells) and ATP (2.24 µmol/mg protein±0.63 µmol/mg protein vs. 1.09 µmol/mg protein±0.61 µmol/mg in control cells; see Table 4). The 5-min incubation in the presence of 10 mM fructose also led to an increase in G 6P levels that was similar to that induced by 10 mM glucose. Notwithstanding, fructose caused a concomitant, significant (P<0.05) decrease in intracellular ATP levels, which reached values of 0.51 µmol/mg protein±0.09 µmol/mg (Table 4). The incubation of isolated hepatocytes with 10 mM glucose+75 µM fructose for 5 min caused a strong increase in G 6P levels, with values about 100% higher than those observed after the incubation with either glucose or fructose alone (Table 4). The combination of glucose and fructose also induced a significant (P<0.05) increase in intracellular ATP levels, although in this case the increase was similar to that induced by the incubation with glucose alone (Table 4).

**Table 4:** Intracellular glucose 6-phosphate and ATP levels and relative increase in serine phosphorylation levels of selected proteins of hepatocytes incubated in the presence or absence of glucose or fructose.

	Control	10 mM Glucose	10 mM Fructose	10 mM Glucose+75 µM Fructose
Glucose 6-phosphate (nmol/mg protein)	16.2±3.4^a^	27.9±4.7^b^*	21.1.±3.0^ab^	51.1±3.0^c^*
ATP (µmol/mg protein)	1.09±0.61^a^	2.24±0.63^b^	0.51±0.09^c^	2.82±0.89^b^
Cyclin A	100±4.9^a^	239.3±5.8^b^*	164.3±5.1^c^	82.3±4.9^a^*
Cyclin B	100±4.3^a^	222.4±5.6^b^*	173.1±4.8^c^	86.5±4.8^a^*
Cyclin D_3_	100±4.0^a^	190.7±5.0^b^*	119.3±4.1^a^	51.2±5.3^c^*
Cyclin E	100±5.1^a^	243.5±6.3^b^*	165.9±5.4^c^	146.2±6.2^c^*
Cyclin H	100±4.9^a^	192.3±6.2^b^*	146.2±5.5^c^	114.9±6.0^a^*
Cdk1-cdc2	100±6.1^a^	192.6±6.9^b^*	137.4±5.3^c^	131.9±5.8^c^*
Cdk2	100±5.5^a^	210.7±6.2^b^*	189.8±5.9^b^	108.8±5.1^a^*
Cdk4	100±5.3^a^	152.1±5.8^b^*	147.3±5.8^b^	114.6±4.9^a^*
Cdk 6	100±4.8^a^	192.7±5.4^b^*	136.4±4.9^c^	106.1±5.4^a^*
cdc 25^a^	100±4.4^a^	147.5±4.9^b^*	107.5±4.3^a^	120.5±5.8^a^*
Cdc6	100±4.3^a^	186.74.8±^b^*	132.2±4.4^c^	119.7±5.6^a^*
AKT 1,2	100±5.3^a^	167.6±5.9^b^*	131.1±5.5^c^	105.2±4.9^a^*
IRAK	100±5.3^a^	241.0±6.1^b^*	172.2±5.0^c^	95.6±4.9^a^*
PYK2	100±5.8^a^	227.3±6.8^b^*	158.5±6.0^c^	100.7±5.0^a^*
PI3 kinase/p85	100±6.3^a^	222.1±6.9^b^*	159.1±5.7^c^	106.1±5.3^a^*
ERK1	100±6.0^a^	180.0±6.6^b^*	136.3±6.3^c^	112.0±5.6^a^*
Total PKC	100±4.9^a^	151.8±5.8^b^	106.4±5.3^a^	142.8±5.9^b^
ERK2	100±4.4^a^	188.0±5.9^b^*	125.3±5.5^c^	116.8±5.3^a^*
PP1,2A,2B, X	100±4.9^a^	188.6±5.1^b^*	115.0±4.4^a^	109.1±5.8^a^*
TRK A, B, C	100±5.0^a^	199.9±5.9^b^*	163.7±5.4^c^	113.3±5.3^a^*
GSK3	100±6.0^a^	149.0±6.6^b^*	114.8±5.8^a^	111.4±5.2^a^*
PTP1	100±5.2^a^	158.8±5.7^b^*	112.2±5.3^a^	99.5±5.7^a^*
PTP1B	100±5.3^a^	134.6±5.9^b^*	102.3±5.9^a^	165.4±6.3^c^*
PTP2	100±6.2^a^	161.3±5.7^b^*	144.7±6.1^c^	79.6±4.6^d^*
Caspase 9	100±5.7^a^	148.7±6.3^b^*	107.7±5.5^a^	102.5±4.9^a^*
c-kit	100±4.7^a^	209.0±5.8^b^*	147.8±4.6^c^	85.7±5.2^a^*
Raf-1	100±4.9^a^	174.7±5.9^b^	107.5±5.0^a^	161.0±5.7^b^
Ras	100±5.8^a^	162.1±6.2^b^*	118.7±5.8^a^	140.8±6.2^c^*
Clathrin	100±5.3^a^	217.2±6.0^b^*	172.2±5.8^c^	90.5±5.0^a^*
Clusterin	100±6.3^a^	150.8±6.4^b^*	118.1±6.6^a^	99.6±4.7^a^*
Hsp-70	100±5.8^a^	153.0±6.3^b^*	142.6±6.5^b^	78.3±4.0^c^*
β subunit insulin receptor	100±5.1^a^	131.0±5.8^b^*	106.2±5.0^a^	99.8±5.1^a^*

Isolated hepatocytes were incubated for 5 min in the absence or the presence of 10 mM glucose or 10 mM fructose, as described in the Material and Methods. The table shows glucose 6-phosphate and ATP intracellular levels, as well as the relative degree of serine phosphorylation of proteins in which differences above 25% were obtained. Results are expressed in arbitrary units, transforming the intensity obtained to 100 in control points. Different superscript letters in a row indicate significant (P<0.05) differences among values from the same row. Thus, data marked with an “a” in superscript are significantly different from those marked with any other letter in the same file. Additionally, superscript asterisks indicate significant (P<0.05) differences between 10 mM Glucose values and 10 mM Glucose+75 µM Fructose ones. In both cases, differences were established following the statistical analysis described in the Material and Methods section. Acronyms for the proteins are described in Figure 1. Results are means±S.E.M. for 5 separate experiments.

**Figure 3:**
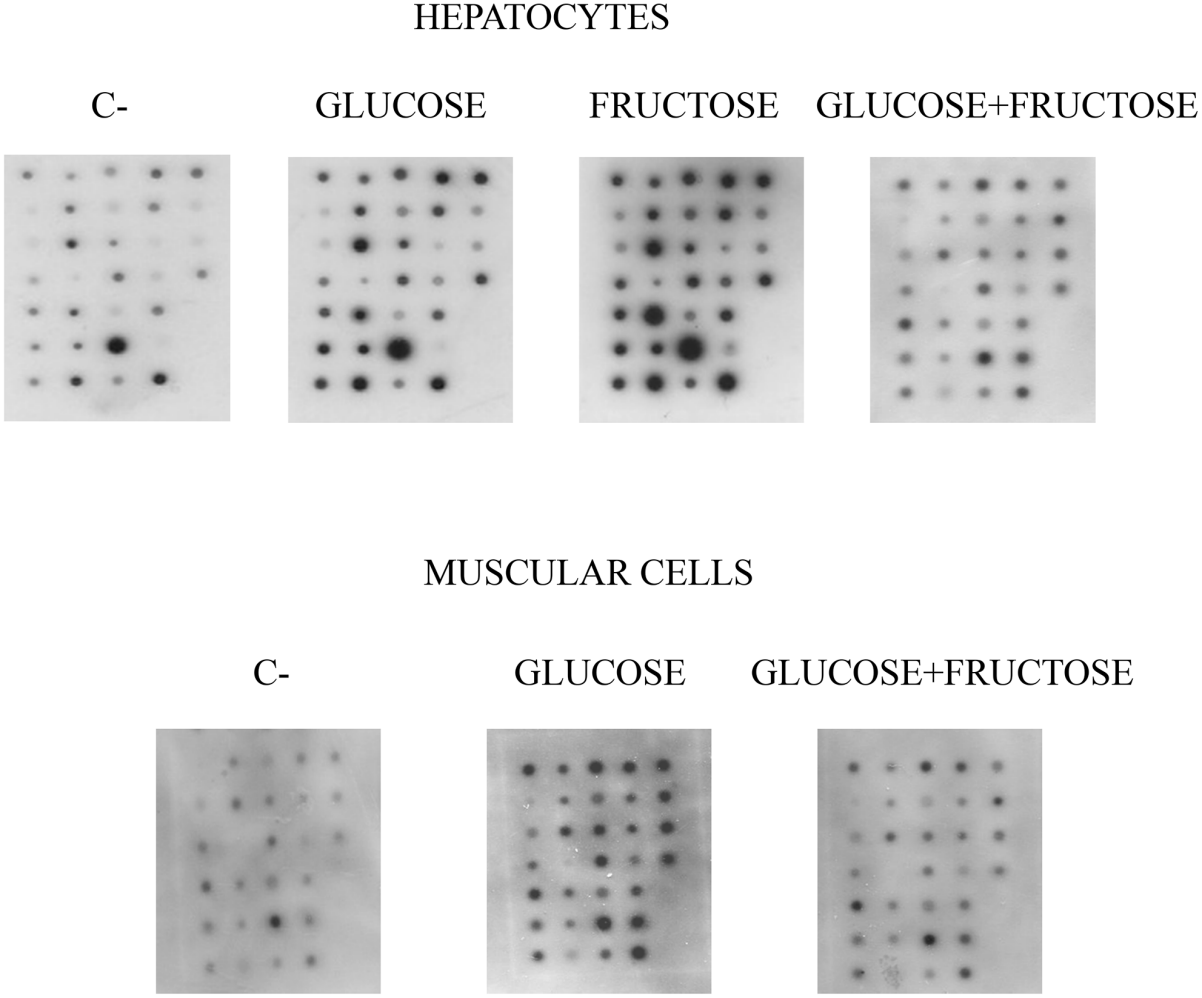
Mini-array analysis of the serine phosphorylation status of several proteins involved in overall cell function and the regulation of cell cycle in isolated hepatocytes and cultured skeletal muscle cells after incubation with a range of concentrations of glucose, fructose or glucose plus fructose. The mini-array analysis and the distribution of the proteins are described in the Material and Methods section and Figure 1. Isolated hepatocytes and skeletal muscle cell cultures were obtained and incubated as described in the Material and Methods section. The serine-phosphorylation levels of each spot in the mini-arrays were then analyzed. Control: cells incubated for 5 min without the presence of any monosaccharide. Glucose: cells incubated for 5 min in the presence of 10 mM glucose. Fructose: hepatocytes incubated for 5 min in the presence of 10 mM fructose. Glucose+Fructose: cells incubated for 5 min in the presence of 10 mM glucose plus 75 µM fructose. Hepatocytes: isolated hepatocytes. Muscle: skeletal muscle cell cultures. The figures shows a representative image for five separate experiments.

Incubation of cells with 10 mM glucose induced a strong and significant (P<0.05) increase in the serine phosphorylation levels of practically all the proteins tested, reaching more than 2-fold increases in protein kinases such as IRAK, PYK2 and PI3 kinase/p85, cell cycle regulating proteins such as cyclins A, B and E and Cdk2, and other proteins, such as c-kit and clathrin (Fig. 3 and Table 4). Remarkably, the effect of the incubation with 10 mM fructose was much less intense. In this regard, there were only three proteins, namely Cdk2, Cdk4 and Hsp-70, in which the increase in serine phosphorylation was similar after incubation with either glucose alone or fructose alone (Fig. 3 and Table 4). In proteins such as cyclins A, B, E and H, Cdk1-cdc2, Cdk6, cdc6, total Akt, IRAK, PYK2, PI3 kinase/p85, ERK-1, ERK-2, total tyrosine kinase, PTP2, c-kit and clathrin, the fructose-induced increase in serine phosphorylation was significantly lower (P<0.05) than that induced by glucose. Furthermore, in contrast to glucose, incubation with fructose did not significantly change the serine phosphorylation levels of cyclin D3, cdc 25A, total protein kinase C, total protein phosphatases (PP1, PP2A, PP2B and PPX), GSK-3, PTP1, PTP1B, caspase 9, Raf-1, ras, clusterin or the β subunit of the insulin receptor (Fig. 3 and Table 4). Remarkably, the incubation of hepatocytes with 10 mM glucose plus 75 µM fructose counteracted practically all the glucose- and fructose-induced increases observed in serine phosphorylation levels (Fig. 3 and Table 4). Finally, the joined glucose/fructose incubation induced a significant (P<0.05) decrease in serine phosphorylation levels of proteins like cyclin D3 and Hsp-70 (Fig. 3 and Table 4).

### Changes in intracellular G 6P and ATP levels and in the serine phosphorylation levels of proteins linked to cell cycle regulation and phospho/dephosphorylation mechanisms in cultured skeletal muscle cells incubated in the presence of glucose or fructose

As in the case of hepatocytes, the *in vivo* experimental design led to the presence of two distinct blood sugar environments for skeletal muscle cells. Thus, after the glucose load, these cells were exposed to a high blood glucose concentration of about 10 mM. After the fructose load, muscle cells were exposed to glucose concentrations around 10 mM, which was accompanied with a concomitant exposure to very low concentrations of fructose (about 75 µM). Given these observations, our next experimental design was based on the incubation of cultured skeletal muscle cells from starved healthy rats in the presence of 10 mM glucose or 10 mM glucose plus 75 µM fructose in order to mimic the range of conditions observed in skeletal muscle cells *in vivo*.

The incubation of these cells in the presence of 10 mM glucose for 5 min induced a strong and significant (P<0.05) increase in the intracellular concentrations of G 6P (from 6.8±1.4 nmol/mg protein in basal cells to 16.8±2.4 nmol/mg protein after glucose incubation) and ATP intracellular levels (from 0.80±0.06 µmol/mg protein in basal cells to 5.12±1.87 µmol/mg protein after glucose incubation, see Table 5). Incubation in the same conditions but with the addition of 75 µM fructose rendered increases in G 6P and ATP intracellular levels that were not significantly different to those observed in the presence of glucose alone (Table 5).

**Table 5:** Intracellular glucose 6-phosphate and ATP levels and relative increase in serine phosphorylation levels of selected proteins from cultured skeletal muscle cells incubated in the presence or absence of glucose or fructose.

	Control	10 mM Glucose	10 mM Glucose+75 µM Fructose
Glucose 6-phosphate (nmol/mg protein)	6.8±1.4^a^	16.8±2.4^b^	19.0±2.5^b^
ATP (µmol/mg protein)	0.80±0.06^a^	5.12±1.87^b^	5.41±1.96^b^
Akt 1/2	100±5.3^a^	198.4±6.6^b^	130.7±5.7^c^
Cdk6	100±5.4^a^	129.0±6.7^b^	87.0±5.0^a^
Cyclin E	100±6.4^a^	169.2±6.6^b^	124.4±5.5^c^
IRAK	100±4.6^a^	164.1±6.9^b^	111.6±5.7^a^
PYK2/CAKβ	100±5.7^a^	178.8±7.1^b^	96.9±6.0^a^
c-kit	100±4.5^a^	90.0±7.3^a^	71.0±5.3^b^
Cyclin H	100±5.8^a^	110.3±6.5^a^	78.3±5.1^b^
PI3 kinase/P85	100±5.8^a^	142.7±7.0^b^	96.3±5.0^a^
c-Raf-1	100±6.0^a^	180.8±6.4^b^	126.7±5.5^c^
ERK-1	100±6.4^a^	156.4±6.8^b^	92.8±5.9^a^
Total PKC	100±6.2^a^	156.5±7.1^b^	100.8±6.2^a^
Ras	100±5.9^a^	194.4±6.0^b^	106.6±5.0^a^
ERK-2	100±6.4^a^	139.5±6.3^b^	80.2±5.1^a^
Total Trk	100±5.8^a^	173.4±6.9^b^	98.9±6.1^a^
PTP1 (SH)	100±5.5^a^	142.1±6.4^b^	102.6±5.3^a^
PTP1 B	100±5.7^a^	163.9±7.1^b^	102.9±5.6^a^
PTP2 (SH)	100±6.3^a^	164.3±7.0^b^	103.7±5.5^a^

Isolated skeletal muscle cells were incubated for 5 min in the absence or the presence of 10 mM glucose or 10 mM fructose, as described in the Material and Methods. Table shows glucose 6-phosphate and ATP intracellular levels, as well as the relative degree of serine phosphorylation of proteins in which differences above 25% were obtained. Results are expressed in arbitrary units, transforming the intensity obtained to 100 in control points. Different superscripts in a row indicate significant differences following the statistical analysis described in the Material and Methods section. Thus, data marked with an “a” in superscript are significantly different from those marked with any other letter in the same file. Acronyms for the proteins are described in Figure 1. Results are means±S.E.M. for 5 separate experiments.

Strikingly, the presence of 75 µM fructose altered the effects on the serine phosphorylation levels shown by glucose alone. Thus, incubation with 10 mM glucose for 5 min induced a significant (P<0.05) in serine phosphorylation levels of a wide array of proteins. The greatest increases were those observed in total Akt (from 100.0±5.3 to 198.4±6.6 arbitrary units), Ras, (from 100.0±5.9 to 194.4±6.0 arbitrary units), c-Raf-1 (from 100.0±6.0 to 180.8±6.4 arbitrary units), PYK2/CAKβ (from 100.0±5.7 to 178.8±7.1 arbitrary units) and total tyrosine kinase (from 100.0±5.8 to 173.4±6.9 arbitrary units, see Fig. 3 and Table 5). However, the serine phosphorylation levels of Cdk6, cyclin E, IRAK, PI3 kinase/p85, ERK-1 and ERK-2, total protein kinase C, PTP 1 and PTP2 also significantly (P<0.05) increased. The incubation with 10 mM glucose plus 75 µM fructose practically prevented all the increases in serine phosphorylation levels induced by glucose alone. Thus, the glucose-induced increases in the above mentioned proteins were blocked by the presence of 75 µM fructose. In addition, the glucose-induced increases in total Akt (from 198.4±6.6 arbitrary units in the presence of 10 mM glucose to 130.7±5.7 arbitrary units in the presence of 10 mM glucose plus 75 µM fructose), cyclin E (from 169.2±6.6 arbitrary units in the presence of 10 mM glucose to 124.4±5.5 arbitrary units in the presence of 10 mM glucose plus 75 M fructose) and c-Raf-1 were partially counteracted (from 180.8±6.4 arbitrary units in the presence of 10 mM glucose to 126.7±5.5 arbitrary units in the presence of 10 mM glucose plus 75 µM fructose, see Fig. 3 and Table 5). Finally, the presence of 75 µM fructose induced a decrease (P<0.05) in the serine phosphorylation levels of two proteins that were not affected by the incubation with glucose alone, namely c-kit (from 100.0±4.5 arbitrary units in control cells to 71.0±5.3 arbitrary units in the presence of 10 mM glucose plus 75 µM fructose) and cyclin H (from 100.3±5.8 arbitrary units in control cells to 78.3±5.1 arbitrary units in the presence of 10 mM glucose plus 75 µM fructose, see Fig. 3 and Table 5).

## Discussion

Our results indicate that glucose and fructose oral loads induce sugar-specific and rapid functional responses not only in liver but also in peripheral tissues like skeletal muscle. The differential effects of these two sugars *in vivo* are easily observed when comparing the results obtained after loads that induced similar increases of glycemia to about 9.5 mM/10 mM and insulinemia to about 200 mIU/L in both cases. In these conditions, with similar glycemia and insulinemia, fructose loading induced a significant increase in serine phosphorylation levels of only muscle PI3 kinase/p85. On the contrary, oral glucose loading increased serine phosphorylation levels of muscle cyclin H, Cdk2, IRAK, total PKC, PTP1B, c-Raf 1, Ras and the β unit of the insulin receptor, without affecting muscle PI3 kinase/p85. This observation indicates that glycemia and/or insulinemia are not the sole factors involved in the differential effects of glucose and fructose on muscle, since these specific effects were caused despite similar values of blood glucose and insulin levels. In fact, that glucose is not the sole factor involved in these specific effects is also highlighted in both isolated hepatocytes and cultured muscle cells. While the addition of 10 mM glucose to the former increased serine phosphorylation levels of practically all of the proteins tested, in the latter the effect was much less intense, and only 15 of the proteins showed an increase. Thus, we propose that other factors are involved in the observed effects of sugars on protein serine phosphorylation.

Regarding the effects observed *in vivo* in liver and isolated hepatocytes, several questions arising from our results. The first concerns the comparison of effects between the two experimental systems. Thus, it is evident that the response to sugars *in vivo* is less intense than in isolated cells. This effect can be caused by several factors, being one of this the unavoidable effect of anaesthesia on both liver and muscle metabolism. However, since the effect of the anaesthesia will be of the same magnitude in all of the experimental conditions studied, we can infer that these compounds will not affect the specific response of the utilized sugars, albeit the intensity of the effects would be changed in a similar intensity in all cases, regardless of the sugar utilized. Additionally, we might highlight that in the case of fructose there are proteins in which the effect is the contrary when comparing the results obtained *in vivo* and in isolated/cultured cells. These distinct effects on whole liver and isolated hepatocytes can be attributed to the experimental systems. In this regard, the treatment by which isolated hepatocytes are obtained modifies their membrane structure, and key membrane proteins like the insulin receptor are altered during this process [19]. This implies that the response to external stimuli, such as sugars, differs between isolated hepatocytes and whole liver. Moreover, the whole liver has a complex structure, in which two types of hepatocytes can be distinguished. The first type, centro-lobulillar hepatocytes, are in direct contact with portal blood and thus are direct targets for all the substances that arise from the portal vein [20]. The other cellular type includes hepatocytes that are in direct contact with the peripheral circulation from hepatic arteria and veins [20]. In these cells, the effects induced by portal substances are mediated through the centro-lobulillar hepatocytes. Thus, a hierarchy of effects induced by either portal fructose or portal glucose is exerted *in vivo*. In contrast, hepatic suspensions mix both types of hepatocyte, thus eliminating any possible hierarchical action of sugars. Thus, the hepatic suspension model will yield results that differ to those observed *in vivo*. This is an unavoidable effect, since the separation of the two hepatocyte subpopulations is not possible in *in vivo* experiments. There are other technical questions that difficult the overall interpretation of the results shown here. Thus, the mini-array analysis can be only interpreted as a broad approach to the specific effects determined in all of the analyzed proteins. This implies that the interpretation of the results obtained with the mini-array technique must be done with caution and a more definitive interpretation of the results would be carried out with the support of complementary techniques like Western blot. However, despite all of these difficulties, the results obtained here are a good indication that both glucose and fructose has specific and separate effects on the overall hepatocyte and myocyte physiology taken as a whole, although the precise ways by which these specificities are launched can not be elucidate at this point.

Despite all these differences and difficulties, the combined analysis of results obtained in *in vivo* and in isolated and cultured cells allows us to draw a number of conclusions. First, the effects of sugars are rapid, since they induce strong changes after only 5 min of incubation in both isolated and cultured cells. Moreover, the different effect of glucose and fructose appears to be associated with the sugar-specific changes observed in the intracellular levels of metabolites like G 6P and ATP. Thus with regard to the liver, the glucose-induced increase in ATP would activate phosphorylation pathways by increasing substrate [9]. Meanwhile, the fructose-induced decrease in ATP would induce the opposite effect [21]. It is noteworthy that the fructose-induced decrease in ATP in hepatocytes is mainly a consequence of the low Km (about 0.5 mM) of FK for fructose, which induces a very high velocity of fructose phosphorylation to fructose 1-phosphate [22]. In fact, the ATP lowering triggered by the fructose-FK system is at the basis of the other well known metabolic alterations induced by this sugar, such as hypertriglyceridemia or the transient increase in serum uric acid [1], [6], [7]. In fact, the mere presence of FK in liver is surprising at a first glance as hepatic cells can uptake and metabolize fructose via other hexokinases, such as the ubiquitous hexokinase-I [23]. Thus FK appears to contribute to modulating the functional status of hepatocytes, which would change their overall functional activity depending on the specific changes induced by the FK pathway on the phosphorylation status of a wide range of proteins. This hypothesis would also explain the presence of FK in the liver of animals whose diets contain low fructose levels, like the dog and cat [24]. In these animals, fructose, via hepatic FK, would serve as a true modulator of hepatic function rather than merely a direct energy source.

However, while the effects of glucose and fructose on liver could be explained by a direct, specific action of these two sugars through their portal door of entry, the effects observed on peripheral skeletal muscle can not be attributed to the same direct effect. In this regard, our results indicate that while glucose alone has a distinct effect on serine phosphorylation of many proteins, fructose alone does not. In fact, hepatic metabolization of fructose implies that, in the best of cases, fructosemia is consistently in the µM range, whereas glycemia is in all cases in the mM range. The sugar-specific effects observed in muscle would be due to an inducer that would be produced from liver after the action of either portal glucose or portal fructose on this organ. We can only speculate regarding this question, although there are several points of interest. First, as indicated above, the sugar-specific actions do not appear to be related to the specific serum glycemia and/or insulinemia, since these effects were observed in conditions in which serum glucose and insulin were similar. We propose that other factors are therefore involved. It is noteworthy that fructose-rich diets cause hyperlipidemia through a hepatic mechanism involving FK and the lowering of intracellular hepatic ATP levels [21]. Thus the increase in lipidemia could be a signalling factor of fructose ingestion for peripheral tissues. Notwithstanding, fructosemia itself is another factor related to the fructose-linked effects in skeletal muscle. A specific effect of micromolar concentrations of fructose can be inferred after analyzing the results observed in isolated skeletal muscle cells, in which the addition of 75 µM of this sugar dramatically changed the glucose-induced effects on protein serine phosphorylation. It is also remarkable that these fructose-linked effects were not associated with changes in intracellular G 6P or ATP contents, since the values of these two metabolites were very similar in skeletal muscle cells incubated with glucose alone or with glucose plus fructose. Regarding this point, there are a series of paradoxes related to *in vivo* fructose utilization. The genetic lack of liver FK induces fructosuria, a benign process in which fructosemia reaches serum concentration values of mM; however, none of the known side-effects related to fructose metabolization, such as hypertension, hyperlypidemia and insulin resistance occur [25]. In fact, one of the very few complications that arise in fructosuria is the appearance of proximal renal tubular acidosis, which is caused by an excess of kidney filtration of fructose [25]. This observation contrasts with the presence of the specific fructose transporter GLUT5 in skeletal muscle [26]. The presence of this transporter indicates that these cells have the capacity to take up fructose from the environment. However, there are no signs that peripheral muscle uses this sugar in conditions of fructosuria. Perhaps this paradox can be explained by the concentration of fructose reached in each condition. In healthy individuals that present hepatic FK, the maximal fructosemia is in the µM range, not mM as in fructosuria. Moreover, this fructosemia is always accompanied by glycemia concentrations in the mM range. This finding is relevant as several studies carried out in isolated hepatocytes indicate that the incubation of rat hepatocytes with 8 mM glucose plus 100 µM fructose caused a dramatic increase in the glycolytic flux by raising the intracellular levels of fructose 2,6-bisphosphate (Fru(2,6)P2) when compared with the incubation with 8 mM glucose alone [27]. The effect of the glucose-fructose combination was accompanied by an increase in intracellular G 6P through a mechanism linked to the glucose-and-fructose induced activation of liver glucokinase [28]. Remarkably, the incubation of isolated hepatocytes with 100 µM fructose alone did not increase the intracellular levels of either Fru(2,6)P2 or G 6P [27]. Thus the induced increase in G 6P levels in hepatocytes incubated with glucose plus fructose cannot be attributed to an increase in the pool of fructose 6-phosphate resulting from fructose metabolism. These reflections raise the question as to the nature of the role played by FK in the modulation of *in vivo* fructose signalling. At present, we can only speculate on this question, and more experiments are required to elucidate the presence of a putative, co-operative action of high glucose concentrations and low fructose concentrations on overall cell function.

**Figure 4:**
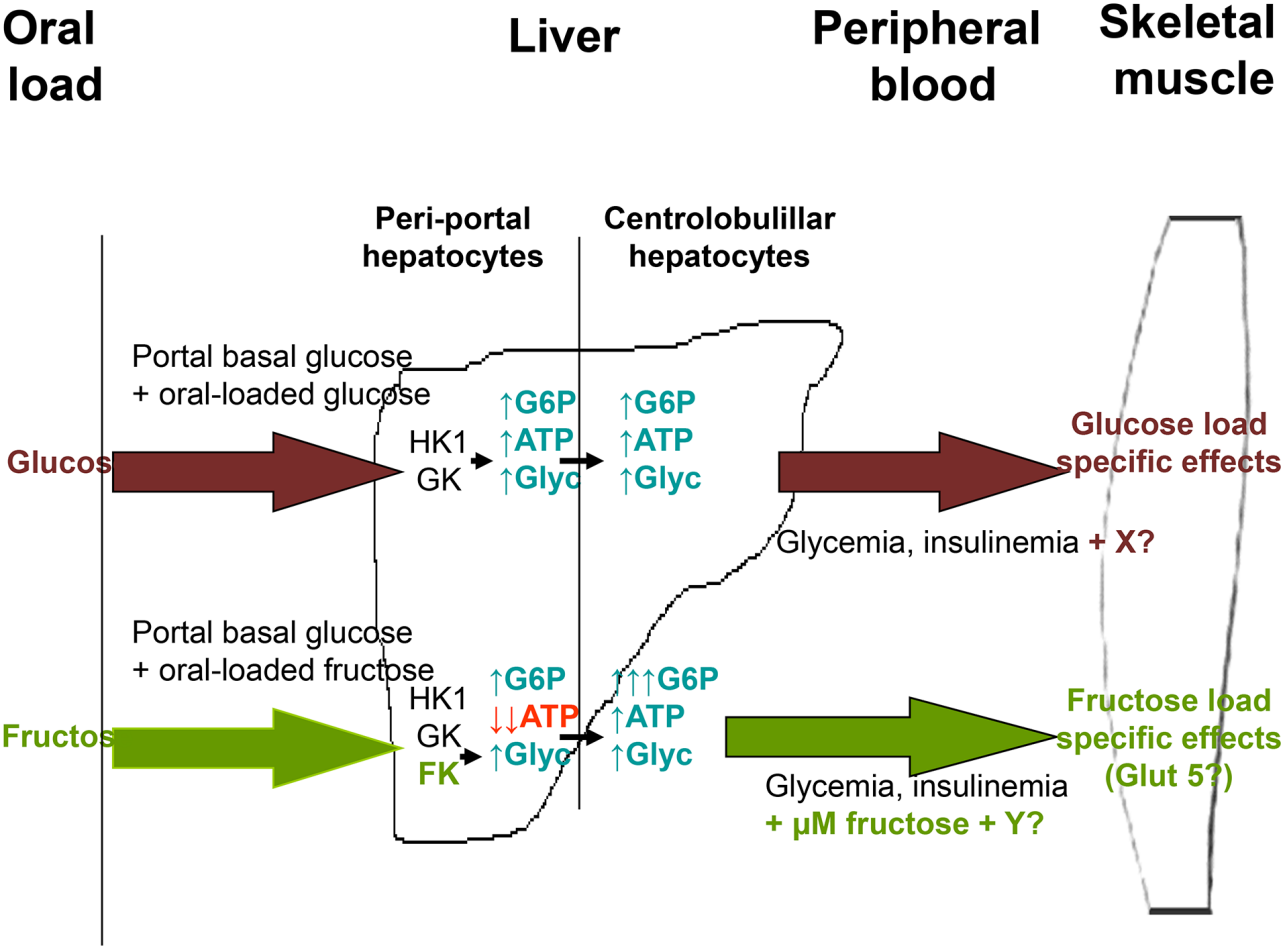
Diagram explaining the postulated hypothesis given to explain the specific effects of fructose and glucose on the overall function of liver and skeletal muscle cells after ingestion of/incubation with these sugars. Following this hypothesis, peri-portal hepatocytes respond to glucose or fructose in a specific manner, depending on the sugar-specific changes that intracellular G 6P and ATP levels undergo in these cells and, perhaps, other unknown signalling mechanisms. The response of these cells is communicated to other hepatic cells and the liver as a whole signals peripheral tissues; this signalling, which is probably based on the presence or absence of micromolar concentrations of fructose in circulating blood, in addition to other putative, unknown signalling mechanisms, indicates that the circulating metabolites derive from either glucose or fructose. Peripheral tissues, like skeletal muscle, would then respond in a specific manner to these signals by changing the phospho-dephosphorylation levels of several key proteins, thus inducing an alteration in the physiology of these cells.

The data collected using our experimental approach allows us to propose a general hypothesis to explain the *in vivo* functional effects of glucose and fructose. In this hypothesis (see Fig. 4), the response of peri-portal hepatocytes to glucose and fructose depends on changes in intracellular G 6P and ATP levels induced by the sugars. This specific response is caused by the presence of separate phosphorylation mechanisms for glucose (hexokinase-I/glucokinase system) and for fructose (FK system). The response of peri-portal hepatocytes is communicated to other hepatic cells and the liver as a whole signals peripheral tissues to indicate the sugar source of the circulating metabolites. These signals may be related to the presence of metabolites such as triglycerides and specifically fructose. Thus peripheral tissues, like skeletal muscle, would respond in a specific manner to these signals by changing the phospho-dephosphorylation levels of several key proteins, thereby inducing alterations in the physiology of these cells. The response of each tissue to the specific signals from the liver would depend on the processing mechanisms involved. For instance, the presence of GLUT 5 in muscle cells would be explained as a specific response mechanism for fructose-induced circulating function modulators. In summary, on the basis of our results, we propose the existence of a general *in vivo* mechanism that utilizes sugars as specific modulators of overall body sugar utilization.
